# A More Biomimetic Cell Migration Assay with High Reliability and Its Applications

**DOI:** 10.3390/ph15060695

**Published:** 2022-06-01

**Authors:** Di Yin, Hongbo Zhang, Chun Yang, Wenjun Zhang, Shihmo Yang

**Affiliations:** 1 School of Mechanical and Power Engineering, East China University of Science and Technology, Shanghai 200237, China; zhizhuxiaoscar@gmail.com (D.Y.); hbzhang@ecust.edu.cn (H.Z.); 2 Department of Mechanical Engineering, College of Engineering, Saskatoon, SK S7N 5A9, Canada; chy416@mail.usask.ca; 3 Division of Biomedical Engineering, University of Saskatchewan, Saskatoon, SK S7N 5A9, Canada; 4 Biomedical Science and Technology Research Centre, School of Mechatronic Engineering and Automation, Shanghai University, Shanghai 200444, China

**Keywords:** cell migration assay, cell patterning, microfluidics

## Abstract

Cell migration refers to the directional movement of cells to the surrounding cell-free zone in response to chemical and mechanical stimuli. A cell migration assay is an essential device for studying pharmaceutical and medical problems. In this paper, we present a novel approach to a cell migration assay on a chip with two merits, namely (i) simultaneous creation of many cell samples on the same condition and (ii) cells migrating while being stressed in a fluidic environment. The first merit has addressed the problem of poor reproducibility in experimental studies for medical problems such as wound healing, and the second merit has made the cell migration device, which is an in vitro environment, more biomimetic. The two merits are attributed to a novel mechanical method to simultaneously create many cell-free zones and to the design of a microfluidic process to create shear stress in cells uniformly. Two applications were studied on our device to explore its effectiveness. The first application is regarding the combination chemotherapy of cisplatin and doxorubicin (Adriamycin) on cervical cancer cells (HeLa). The second application is regarding inhibiting the migration of endothelial cells (HUVEC) in the process of anti-angiogenesis.

## 1. Introduction

Cell migration refers to the movement of cells from one location to another under various stimuli, including shear stress [1], topography [2], oxygen [3], cytokines [4], and extracellular matrix proteins [5]; it is considered as one of the important indicators of the physiological and pathological process, e.g., wound healing [6,7,8,9,10]. There is evidence that during wound healing in tissues, cell migration is essential for the re-epithelialization of the skin [11,12]. However, studies of cell migration in vivo are limited by ethical issues with animal experiments, and thus are only possibly conducted in vitro with engineered devices that mimic the in vivo environments. This calls for the development of devices that can mimic the in vivo environments. Several devices have been developed for a wide range of screening applications, such as toxicity and viability studies [13,14].

Furthermore, a fluidic environment is indispensable for any of such biomimetic in vitro devices [15], where cells are thus under shear stress. There is a report that cells respond to shear stress by activating ion channels [16], gene expression [17], and reorganization of the cytoskeleton [18]. Studies have also shown that cells of different types migrate significantly differently in organ environments that have different biochemical and physical properties [19,20,21].

Devices for cell migration experiments are required to allow cells to move to a so-called cell-free area. There are two design concepts for meeting this requirement: the transwell migration assay and wound healing assay [22]. The former is to measure the number of cells passing through a microporous membrane within a certain time. This approach is used as a standard one for quantifying the cell migration ability in the medical industry, also called Boyden, or the modified Boyden chamber assay. However, this approach has an inherent limitation that it cannot monitor the cell migration process in a fluidic environment and in real-time. The latter overcomes this limitation by measuring the rate that monolayer cells enter engineered cell-free zones (CFZ) while cells are being shear stressed [23,24].

There are two methods to create CFZ, namely cell exclusion and cell depletion. The cell exclusion method is to limit the area of cell growth by using blocks, solid barriers, and liquid barriers, and this method has the benefit of little cell damage [25,26]. The cell depletion method is to remove cells in a region by scratching, stamping, heating, laser ablation, biochemical effects, and sharp objects, and this method has little physical damage on the cell surface [24,27,28,29,30,31]. Of them, the scratch method is the most common one because of its simplicity and low cost [29]. The common technique used in the scratch method is that the operator uses pipette tips to create a blank area in monolayer cells [32]. As such, the scratch method has low reproducibility, making the experiment of cell migration unreliable.

In summary, a device that mimics an organ environment for cell migration is expected to meet the following requirements: (1) be able to provide different fluidic environments suitable to different types of cells; (2) be able to monitor the movement of cells in real-time [33]; (3) be able to produce many samples in the monolayer cells under the same condition. It is worth mentioning that the requirement (3) is completely neglected in literature, the reason of which is perhaps the dogma among researchers that to get many samples, one only needs to repeat the same experiment many times. However, this dogma is questionable to biological systems due to their highly sensitive nature to time and space and; thus, they are highly variable, notwithstanding a possibly high cost by repeating the experiments in many times. For drug testing on cells, production of a few hundred samples under the same condition is very important to the reliability of drug testing.

At present, there is no commercial product and device in a public domain that fully satisfies the above requirements. The Oris™ Cell Migration Assay is a commercial test platform, which is based on 96-well plates. Each well represents an independent experiment condition. However, this device fails to meet the requirements (1) and (3), mentioned above. In literature, Lin et al., used the trypsin flow-focusing technique to form a long strip of CFZ for investigating the anti-invasive effects of the drugs [24]. Unfortunately, their device failed to meet the requirement (3), besides potentially a considerable level of cell damage. Further, in their work, CFZs are highly variable due to a manual process. In [34,35], a method to use trypsin to create the CFZ was developed as well, but the method adds uncertainty regarding cell dynamics.

To fully meet the above-mentioned requirements, we have developed a novel device by using the microfluidic technology. Our device can create many samples (around 400 samples) under the same condition and in a uniform shear stress environment during the cell migration process. To ensure the consistency of the samples of cells in terms of shear stress in them, a computational method is employed to obtain the information of shear stress in cells, which is used to select qualified cells (in terms of shear stress). To overcome the problem of poor reproducibility in literature, the creation process for CFZs is made automatic. Two studies were conducted on our device, which are a combination of chemotherapy and anti-angiogenesis. In short, our device has the following salient features: (1) creation of a fluidic environment, thus it is more biomimetic; (2) free of the reproducibility problem in sample preparation; (3) less damage to cells; (4) creation of many samples (around 400 samples in this case) on the same condition; (5) the process of creating CFZs being automated.

## 2. Results

### 2.1. Design Principle for the Microfluidic Chip

**Figure 1:**
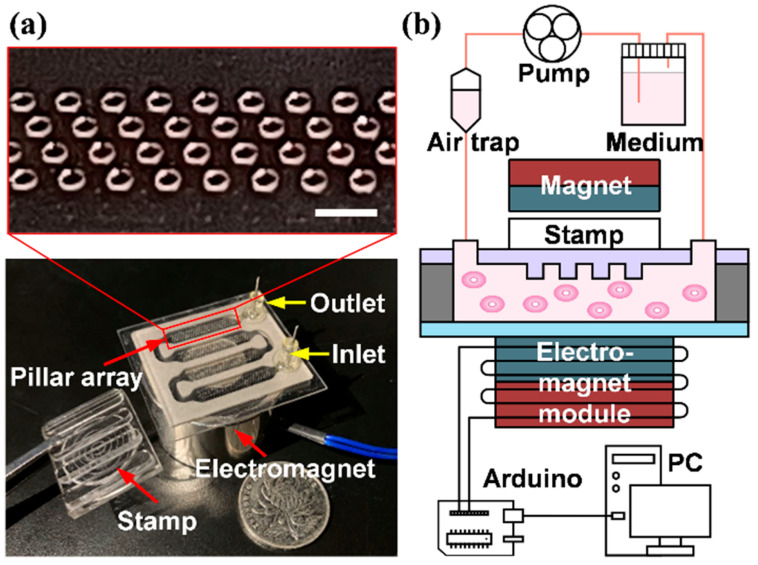
(**a**) The chip with the cell culture chamber in our system. The inset shows a partial enlargement of the micro-pillar array in a dark background. Scale bar, 1 mm. (**b**) The schematic illustration of the overall equipment includes the perfusion system providing the fluid environment, the microfluidic chip for cell culturing, and the electromagnet system controlled by a microcontroller (Arduino) and a personal computer (PC).

Our device is shown in Figure 1. It is illustrated in Figure 1b that the micro-device has achieved three functions: (1) cell culturing, (2) resettable stamping, and (3) a shear stress environment. The devices had four cell culture areas with a size of 105 mm^2^, and 400 CFZ per operation. The size and space utilization of the chip was far superior to the cell migration (wound healing) assay products commercially available, which are based on the industry-standard 96-well format [36]. The elastic PDMS was selected as the material for the micro-pillar layers, which keep no deformation while stretching the PDMS substrate, thus ensuring the well-defined pattern of cells; this also ensured a minimal physical damage to the culture substrate (Figure 1b). However, the elasticity of the PDMS may cause the pillar to deform while contacting the bottom surface, thereby affecting the size of the cell-free zone. Departing from the devices reported in literature, which is a manual process, in our device, the process was automated, especially since an electromagnet system was used to drive the stamping process. To provide a stable shear stress environment, a long and narrow chamber are indispensable, but height is limited by the pillars. If the pillars are too close to the bottom of the chamber, they will interfere with the flow field in the environment where the cells are located and make it difficult to obtain the same magnitude and direction of shear stresses. Through trial-and-error, with the help of simulation, it was found that the height of 0.8 mm will not produce the said interference. To ensure the stability of the flow field, the cavity needs to maintain a fixed shape, and the bottom of the cavity is made of a petri dish, which not only facilitates cells’ attachment but also has a certain degree of stiffness. Due to the low stiffness of the PDMS at the top layer of the chip, obvious bulges would be generated during the shearing process. To prevent this deformation from disturbing the fluidic environment in the channel, the height of the channel was restricted by a simple fixture. By the way, our device did not take the architecture that has a multi-layer cavity with PDMS film in [26].

### 2.2. Characterization of Stamping Controlled by the Electromagnetic System

**Figure 2:**
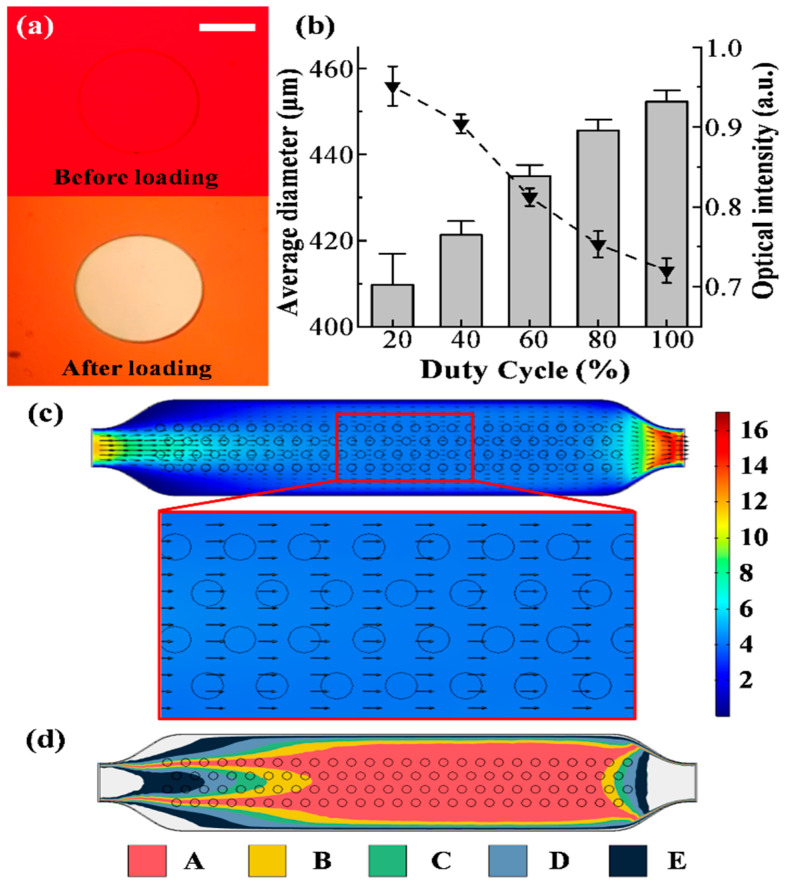
(**a**) Micrographs of the micro-pillar before and after loading stamp. Scale bar, 200 μm. (**b**) Diameter created by stamping under a different duty cycle of the PWM wave in the electromagnet system, which generates magnetic force. The height of the chamber for different loads that were regulated by PWM obtained with the optical intensity measurement. (**c**) Distribution of velocity on the bottom of the chamber while inlet flow rate was 10 mL/min via the simulation. The direction of the arrow represents the flow direction of the fluid at the point. (Unit of scale: dyne/cm^2^) (**d**) The relative error distribution of the shear stress on each cell-free zone at the bottom in conditions of a 10 mL/min inlet flow rate. (A, B, C, D, and E represent areas less than 10%, 20%, 30%, 50%, and 80%, respectively) via the simulation.

Regarding the process automation, our approach differs from others, e.g., by the deployment of gas valves, which has the problem of low sample size [25]. In our device, a controllable electromagnetic system was developed. The requirements for this electromagnetic system can be summarized as follows. First, it shall provide a sufficient and stable force to stamp micro-pillars down toward the culture dish substrate. Second, it shall maintain a precise control of the force to ensure the deformation of micro-pillars is tunable. To access the feasibility of equipment and configuration, an experiment was set up for investigating the effects of the electromagnet system by filling a red edible pigment into the micro-chip and measuring the contact surface between the micro-pillars and the substrate during the loading process. When the PDMS layer was forced to move downward, the pigment right below micro-pillars would be pushed out, resulting in the formation of a series of well-defined circular boundaries. Figure 2a demonstrates the alteration of hue in the contact area before and after loading. It can be observed that after the stamp was loaded, the boundary remained a circular shape, but the diameter became a little larger than before. As the duty cycle of the PWM increases, the electromagnetic force increases, leading to the increase of diameter of the light-colored circle, which was measured by AOI tools in Image-Pro plus, as shown in Figure 2b. When the duty cycle of the PWM was below 20%, the pliable PDMS layer deflected towards the substrate until micro-pillars reached the surface of the substrate. When the duty cycle was higher than 35%, micro-pillars completely expelled the ink and formed distinct circle regions. Under a 40% duty cycle condition, the diameter of the circle remained 422 μm since there was a slight deformation of micro-pillars. When loading above 40%, the diameter showed an increasing trend but a slow upward trend. Pigment in the rest of the undesignated regions began to be expelled out when loading above 90% because the height of the chamber was compressed with increasing weight. Hence, 40% was chosen for all subsequent experiments. In addition, the pixel gray value showed that even at a 100% duty cycle, there was still more than 70% space for cells to pass through.

### 2.3. Simulation Study of Shear Stress in the Microfluidic Chip

Since the cavity of the chip is not a common parallel plate channel, the pillar array above the cavity may cause interference to the flow field at the bottom, and the flow field in the chip also has the problem of uneven distribution. Therefore, it is necessary to simulate the flow field at the bottom of the chamber in order to investigate the shear stress on cells.

As shown in Figure 2c, the line frame and circle in the figure represent the cavity contour and micro-pillar array on the PDMS layer, while the colored part represents the flow field at the bottom of the chamber. Partial enlargement in Figure 2c indicated that on account of the design of the channel, there was a wide area presenting the same direction of fluid flow on the bottom of the chamber, which proved that the pillar array did not affect the flow direction of the fluid at the bottom surface with 0.8 mm of the chamber’s height, and the direction of the arrow also represented the direction of the shear stress on the bottom. The simulation results also showed the flat stress profile except for the region close to the inlet and outlet. To meet the requirements of creating a proper level of shear stress in the cell, particularly the average shear stress on the bottom surface being 4 dyne/cm^2^ and 1 dyne/cm^2^, the inlet flow rates were calculated with the simulation system, and they are 10 mL/min and 2.6 mL/min, respectively. By the way, these parameters will be used for the subsequent simulations and experiments. At the same inlet flow rate, the shear stress in the parallel plate model can be calculated from the equation as follows:

(1) $$\pi =6\mu \frac{Q_{c}}{w_{c}h_{c}^{2}}$$

where *τ* is the shear stress, *μ* is the fluid viscosity, *Q_c_* denotes the flow rate, and *w_c_* and *h_c_* are the width and height of the culture channel, respectively [37]. The results calculated by Equation (1) with the inlet flow rate of 10 mL/min and 2.6 mL/min are 3.23 dyne/cm^2^ and 0.81 dyne/cm^2^, respectively, with a difference of 19.25% and 19.00% from the simulation results. It can be seen that the structure of the flow chamber makes the shear stress at the bottom of the chamber greater than the theoretical calculation of the common parallel plate. In summary, each CFZ at the bottom of the cavity bears the same direction of shear stress, but the magnitude is different. In order to quantify the difference in shear stress in each area, the whole area is divided into several parts based on the relative error range, and it was expressed in different colors. Figure 2d shows the shear stress distribution when the average shear stress is 4 dyne/cm^2^ and the relative error values are 10%, 20%, 30%, 50%, and 80%. As can be seen from the figure, to improve the uniformity of the shear environment of selected samples and ensure the reliability of the result, only samples with their relative error of shear stress being less than 10% should be chosen. This means to select samples from 66 CFZs within district A (red). With the decrease in the inlet flow rate, the CFZ in district A will gradually increase. When the inlet flow velocity is 2.6 mL/min, the average shear stress is 1 dyne/cm^2^. Except for the 11 CFZs near the exit and entrance, the shear stresses in the district A are close to the average shear stress. As such, samples in this situation are considered consistent. In conclusion, the larger the flow rate at the chip inlet, the more uneven the shear stress distribution in the region. However, with the assistance of the simulation software, the samples with higher homogeneity in the mechanical environment can still be selected without compromising the benefit of reliability with the environment.

### 2.4. Microfluidic Cell Migration Assay

**Figure 3:**
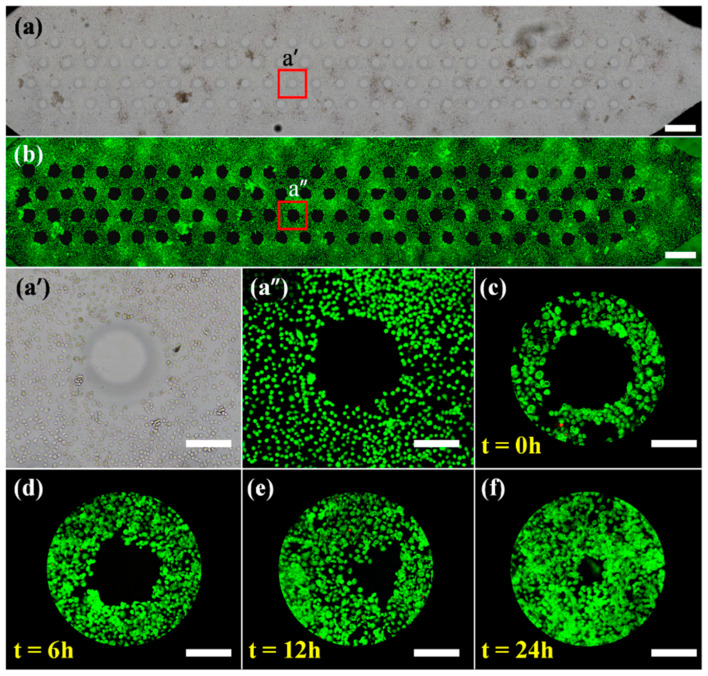
Scan images of (**a**) bright field and (**b**) fluorescence illustrate 100 CFZs in one of four chambers. Scale bar, 1 mm. (**a’**) Bright-field and (**a″**) fluorescent micrographs are partial enlargements of the same red spot in (**a**,**b**), which shows that areas made in our device had almost no dead cells. (**c**–**f**) Time-lapse fluorescent images of cell migration in the microfluidic channel. Scale bar, 200 μm.

Our device guarantees the information-rich readout in the process of quantifying cell migration, improves the reliability of the results, and solves the reproducibility problem with scratch-free methods. To verify the effect of cell patterning, the bright and fluorescent fields of CFZs were scanned, as shown in Figure 3a,b. It can be clearly seen that all the 100 CFZs had well-defined boundaries. Compared with the number of living cells, there were only a few dead cells in the fluorescence image. These two patterns also illustrated that the imaging of cells on the substrate would not be affected by the micro-pillars above them. By zooming in on the bright-field (a’) and fluorescence (a″) images of the same spot in the scan, only one or two dead cells were identified at the edge of the CFZ, suggesting that the creation of CFZ in our device would not be affected by the dead cells.

To further verify whether the cell migration process is affected with the cell depletion method, the time-lapse images of HeLa illustrated the CFZ situation during cell migration from 0 to 24 h are shown in Figure 3c–f. Only a handful of dead cells have been found before and during the CFZ’s creation. The migration area reached 98.2% of the initial cell-free zone at 24 h. There was no significant difference from the migration rate in the cell exclusion method.

### 2.5. Shear Stress and Drug Effect on Cell Migration

**Figure 4:**
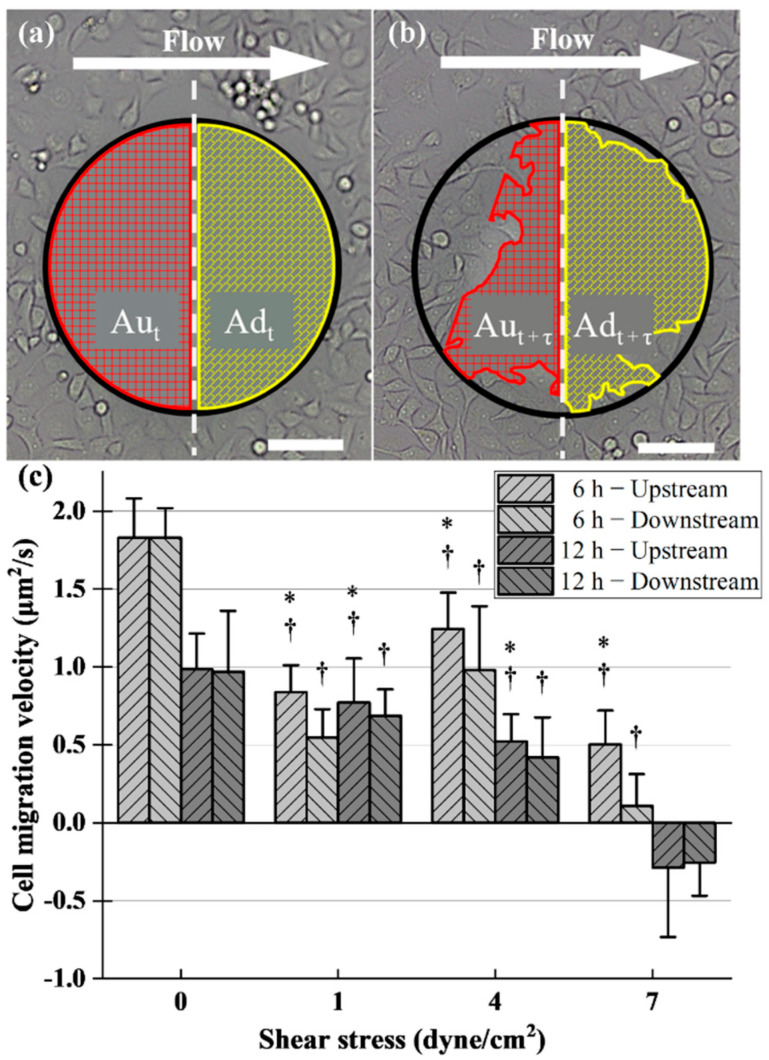
Micrographs of CFZ are divided into two areas from the middle, namely the upstream region (red) and the downstream region (yellow). The areas of these two regions are denoted by Au and Ad, respectively. (**a**,**b**) indicate the areas of different time points t and t + τ during cell migration. (**c**) shows that cell migration velocity along both CFZ regions at 6 h and 12 h under static conditions, low shear stress (1 dyne/cm^2^), middle shear stress (4 dyne/cm^2^), and high shear stress (7 dyne/cm^2^). The results are derived from measurements on 20 CFZs from four chambers. † Statistically significant difference relative to corresponding static control, *p* < 0.01. * Statistically significant difference relative to corresponding “downstream” condition, *p* < 0.01.

To measure the pattern and speed of cell migration under different shear stresses, CFZ was divided into two regions from the middle and defined as upstream and downstream regions, as shown in Figure 4a. The direction of cell migration in the upstream region was the same as the direction of fluid flow, while the direction in the downstream region was the opposite. At t hours, these two areas are represented as *Au_t_* and *Ad_t_*, and after *τ* hours, the areas are expressed as *Au_t+τ_* and *Ad_t+τ_*, as shown in Figure 4b. In *τ* hours, the rate of cell migration in both regions can be estimated by:

(2) $$V_{u}=\frac{Au_{t}-Au_{t+\tau }}{\tau }$$

(3) $$V_{u}=\frac{Ad_{t}-Ad_{t+\tau }}{\tau }$$

With the two equations above, we calculated the migration rates of HUVEC under different shear stresses at 6 h and 12 h, as shown in Figure 4c. The figure illustrated that in a static environment, the cell migration velocity in 6 h is about 1.9 times than that in 12 h. With the increase of shear stress, the inhibition of cell migration within 6 h was enhanced. When the shear stress was 1 dyne/cm^2^, the migration velocity of cells became half that of the control group, and then the inhibition effect was weakened. At 4 dyne/cm^2^, the migration rate reverted to 57.4% of the no-shear environment. After that, the inhibitory effect was strengthened again; even the cells exfoliated, which led to the minimum migration velocity at 7 dyne/cm^2^. On the other hand, the inhibitory effect of shear stress on cell migration increased with the increase of shear stress at 12 h. The maximum velocity is about 1 μm^2^/s in absence of the shear condition, and the minimum velocity is negative, which appeared at the shear stress of 7 dyne/cm^2^. At this time, the cell exfoliation made in the migration could not be carried out normally, leading to the larger CFZ area. In addition, Figure 4c also illustrated the magnitude of the downstream and downstream velocities. In a no-shear environment, there was no difference in the speed of the upstream and downstream migrations during their respective periods. Under the shear stress of 1 and 4 dyne/cm^2^, the migration velocity of the upstream region was faster than that of the downstream region, and the difference between the two speeds was greater in 6 h than in 12 h. In the condition of 7 dyne/cm^2^, the migration rate of the first 6 h is still higher in the upstream region. At 12 h, the velocity of both regions became negative. Not only did the CFZ area become larger, but the surrounding cells also peeled off and the cell density decreased.

**Figure 5:**
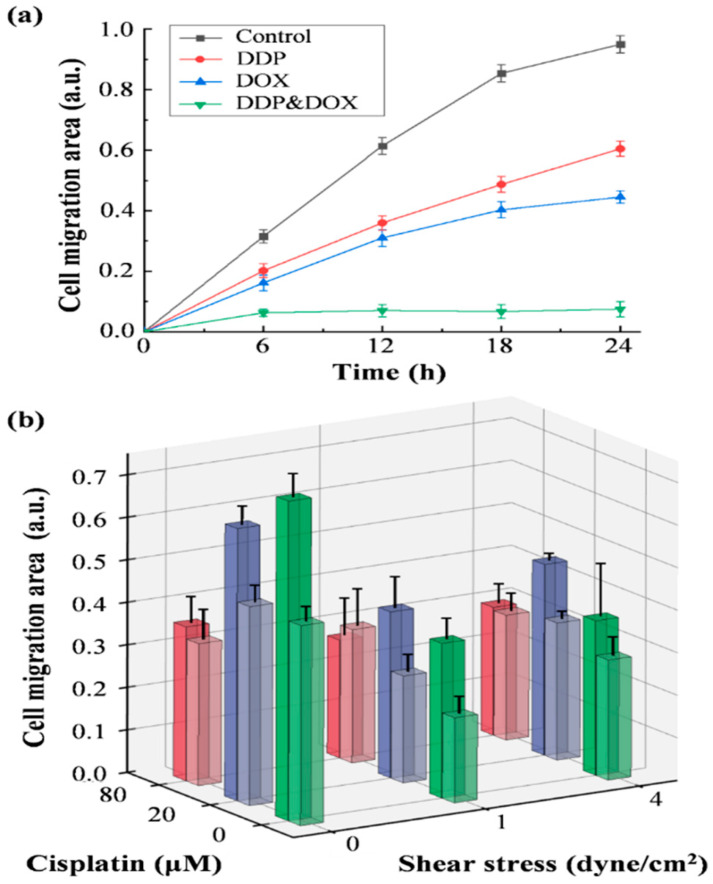
(**a**) Cell migration area of HeLa in 24 h influenced by DDP, DOX, and their compounds vs. the control group. (**b**) Cell migration area of HUVEC under different shear stress and concentration of cisplatin at 6 h (light color) and 12 h (dark color) (*n* = 20).

The device was also used for investigating combination chemotherapy that treats cancer cells with more than one medication at a time. In our study, the HeLa cell line was used to study the efficacy of combination chemotherapy of cisplatin (DDP) and doxorubicin (DOX). Both drugs can induce apoptosis and growth arrest of HeLa cells by interacting with DNA, resulting in weakening of cell migration. According to the instructions, DDP and DOX were diluted into 2 μg/mL with their suitable solvents, respectively. Single and multiple drugs were injected into the chip during cell migration, and the migration area was photographed every 6 h. The cell migration areas of Hela cells are shown in Figure 5a. The different trend of curves of treatment groups and the control group showed that the HeLa cell is sensitive to both drugs. The fastest migration was in the control group, where HeLa cells healed about 95.0% of the area in 24 h. The moderated increase of the cell migration area after adding inhibitors indicated that the cell mobility was significantly reduced. In the experimental group supplemented with DDP and DOX alone, the cell migration rate was similar in the first 6 h, and the corresponding area was 20.2% and 16.2%, respectively. Over time, the difference in cell migration between the two groups treated with different drugs became larger. At 24 h, the migration area was 60.5% and 44.5%, respectively. This shows that DOX presents a more impeding effect in cell migration compared to DDP. The cells in the DDP&DOX group, on the other hand, healed only 6.29% in the first 6 h, and this area remained almost the same over the rest of the time, which means that this compound completely restrains cell migration. It also suggested that as broadly used inhibitors, their combination can achieve a more compromising therapeutic effect.

To investigate the cell migration under the combined influence of shear stress and drugs, a set of experiments was performed for studying the inhibitory effect of cisplatin on the formation of new blood vessels. It is well known that cancer cells need blood vessels to feed their growth, and researchers have developed a series of chemotherapy treatments that indirectly inhibit the growth of cancer cells. Compared with chemotherapy that acts directly on cancer cells, endothelial cells, as the target of antiangiogenic therapy, are genetically more stable than cancer cells and are less likely to develop drug resistance [38]. Cisplatin is a commonly used chemotherapeutic agent, which can reduce matrix metalloproteinase (MMPs) activity of endothelial cell, and the MMP is the main factor regulating the cell migration [34]. In order to simulate the microenvironment of blood vessel formation in vivo, mechanical shear stresses of 1 dyne/cm^2^ and 4 dyne/cm^2^ were introduced into the cell culture environment [29]. After using the cell depletion for creating a CFZ, the shear stress was immediately applied and maintained throughout the cell migration assay. We compared the cell migration area at 6 h and 12 h in the environment with different shearing forces and drug concentrations, as shown in Figure 5b. The migration area at 6 h was represented by light-colored columns, among which the migration rate was the fastest in the control group without drugs and without shear stress, and the slowest in the group with only 1 dyne/cm^2^ shear stress, which was only 43% of the control group. The migration area at 12 h is indicated by dark columns, in which the groups with the largest and smallest migration area were the control group and the group under 1 dyne/cm^2^ shear stress and 80 μM of the drug, respectively. Under the same shear stress, in the first 6 h, the migration rate of cells in the drug-only environment decreased with the increase of the drug concentration, and this trend remained until 12 h and later. At the shearing force of 1 dyne/cm^2^, the situation was quite different, and the cell migration rate increased with the increase of drug concentration. At 12 h, the migration area of the two groups without the drug and with 20 μM of the drug increased and maintained the same relative relationship as that of 6 h, while the migration area under the influence of 80 μM of the drug became smaller than that of 6 h. Under the condition of 4 dyne/cm^2^, the fastest and the slowest cell migration rate at 6 h were the two groups with 20 μM of the drug and without the drug, respectively. At 12 h, the group with the largest migration area was still the group with 20 μM, while the group with the smallest migration area became the group with 80 μM. From another perspective, in the case of the same drug concentration, with different shearing forces, the migration area also had a different trend. When the concentration reached 20 μM, the migration rate of the group with a shear stress of 1 dyne/cm^2^ was the slowest, and that of the group without shear stress was the fastest, which was consistent with the relative relationship of the migration area in the absence of drugs. However, when the concentration reached 80 μM, the migration rate of the cells decreased with the increase of shear stress in the first 6 h. After 12 h, the group with the largest migration area was still the group without shear, but the minimum migration area became 1 dyne/cm^2^, which was even smaller than its area in 6 h. These results indicated that the inhibition effect of shear stress and drugs on HUVEC was not simply superimposed. Under certain conditions, they would even weaken each other, resulting in a certain recovery of cellular migration capabilities.

## 3. Discussion

The cell migration assay is widely used in wound repair, immune response, cancer, vascular disease, and other studies. To further simulate the real situation in the human body in vitro, it is essential to build a bionic environment, and one of the aspects that is often overlooked is that biomechanics affects every tissue and organ all the time. In addition to integration and automation, the new diagnostic tools also need to be more biomimetic, which will help researchers better understand the processes and mechanisms of cell migration and promote the widespread use of the cell migration assay. Therefore, we developed a microfluidic device with a simple three-layer structure that is cell-friendly and similar to a parallel plate channel. Based on this chip, we have developed two methods to create CFZs which are not affected by the dead cells. One is the traditional micro-stencil approach, and the other is the combined method using stamping and chemical treatment, which has been proven to be very effective. Furthermore, samples are selected with the aid of simulation software to ensure the uniformity of the shear environment. These characteristics have greatly improved the performance, reliability, ease of use, and robustness of the cell migration chip. The pattern and speed of cell migration under different shearing stresses were also evaluated. We found that in the first 6 h, shear stress had different effects on cell migration velocity in the upstream and downstream regions. At the same time, the usefulness of the chip to the research of a combination of chemotherapy and anti-angiogenesis has also been demonstrated. The migration ability of HeLa was inhibited by DDP and DOX. The combination of these two drugs completely impeded the cellular migration ability, which achieved the effect that the whole is greater than the sum of the parts. In addition, the cell migration of HUVECs in the first 12 h showed that the inhibitory effects of shearing stress and drugs on angiogenesis would be different at different time points. Even in the early period, the rate of cell migration did not decrease with the increase of drug concentration in the shear stress environment, which differs from cell migration in a purely pharmaceutical setting. The result further reveals that the effects of drugs on cell migration in a fluidic environment (similar to in vivo) is different from in vitro (no-shear environment).

However, there are a couple of limitations with our present work. First, the information on pH has not been monitored, but this information not only affects the cytotoxicity of the pH-dependent drugs such as DDP and DOX [35], but it also alters the activity of proteases such as MMPs, which are the main factors regulating cell migration [39,40]. Specifically, in our present device, a large volume reservoir of the perfusion system is set up to maintain stable alkalinity. In the near future, we will put sensors to measure the extracellular pH (pHe) and intracellular pH (pHi), which are feasible with film sensors [41]. Second, more cell lines, specifically non-cancer cells (e.g., keratinocyte or fibroblasts epithelium) related to the wound healing process will be attempted with our device in the future, though our device is developed without a constraint to a specific type of cells; indeed, cell migration is a feature with any type of cell.

In short, our integrated, automated micro-device has the characteristics of further functionalization and a wide range of promising applications, including (a) the cycle of shear stress experiments on cells, which further simulates a more complex bionic environment and drug therapy; (b) migration studies of different cell types on different coating substrates; (c) a co-culture for a quantitative study on in vitro cancer metastasis and invasion; (d) portable experimental equipment with additional automated modules; (e) investigation of the cellular phenotype and genetic information; and (f) construction of a high-throughput drug screening platform.

In the future, our effort will be taken to (1) making the device self-healing and robust; (2) making an alternative sensor for the device, instead of the image-based sensor, e.g., nanofilm sensors [41]; (3) developing a new robotic micro-fluidic device for shearing force management; (4) monitoring the environmental parameters such as pH and temperature induced via UV light; and (5) constructing a more biomimetic environment with more physiological parameters to be measured and, thus, more readily tailored to specific pharmaceutical applications [42].

## 4. Materials and Methods

### 4.1. Design and Fabrication of Device

The microfluidic device in our system consisted of three layers, which were the PDMS layer with micro-pillars, double side tape spacer, and culture substrate. The PDMS layer was fabricated with a standard lithographic process. The mold, made of negative photoresist (SU-8) with a 100 μm deep micro-well array, was solidified on a silicon substrate after exposure and development. Next, the PDMS (Dow silicone and encapsulants, Sylgard 184 silicone elastomer kit, Dow Corning, Midland, MI, USA) was mixed in a proportion of 10:1 and vacuumed. The degassed mixture was poured onto the mold and cured at 80 °C for an hour. Then, the cured PDMS layer with a micro-pillar array was prepared and separated from the mold. The array contained four regions and each one consisted of 100 cylinders with a 400 μm diameter and a 100 μm height over around 3.5 mm × 20 mm areas. The sidewall of the channel made by double side tape (VHB-4910, 3M, St. Paul, MN, USA) contains four connected chambers that were fabricated by stamping. Each chamber’s size is 21 mm × 5 mm × 0.8 mm. The culture dish (430167, Corning, New York, NY, USA) was used for the substrate, which was conducive to cell attachment, spreading, and migration. Prior to assembly, holes on the PDMS layer were manually punched for inlets and outlets by using a 1.25 mm diameter biopsy puncher (World Precision Instruments, Sarasota, FL, USA). Besides, PDMS was treated with O_2_ plasma to promote the performance of bonding. A double-sided tape was first stuck on the culture dish and then aligned and attached by the treated PDMS layer. The connectors were inserted into the inlet and outlet holes and joints were sealed with epoxy; Figure 1a. The chip was kept in a sterile environment for further experiments.

In addition to the chip, the whole device also includes a perfusion system and an electromagnet system, as shown in Figure 1b. The perfusion system connected to the inlet and outlet of our microfluidic chip was placed in the incubator to maintain the temperature and to keep the cell viability. The values of shear stress covered by our device are from 1 to 7 dyne/cm^2^, which is the approximate range of the shear stress in veins [43]. The culture medium was stored in a reservoir and driven by a high-flow, low-pulsation peristaltic pump. The air trap and soft polyvinylchloride extension tubes between the pump and microfluidic chip further alleviated the pulsatile flow. To improve the reproducibility of the stamping process, the magnet and stamp placed above the chip are combined with the controllable electromagnet module (EM) below. Through the PWM signal sent by the Arduino and PC, the stamping force can be adjusted accurately.

### 4.2. Cell Culturing and Fluorescent Image Acquisition

HeLa cells and Human Umbilical Vein Endothelial Cells (HUVEC) were received from Shanghai Key Laboratory of Orthopaedic Implants, Department of Orthopaedic Surgery, China. They were cultured in Dulbecco’s modified Eagle’s medium (DMEM, Gibco, Thermo Fisher Scientific, Waltham, MA, USA) with 10% fetal bovine serum and 1% Pen/Strep (Gibco, Thermo Fisher Scientific, Waltham, MA, USA) in humidified conditions, which maintained a temperature of 37 °C and a CO_2_ level of 5%. When 80% confluence was reached, cells were harvested with trypsin/EDTA (Gibco, Thermo Fisher Scientific, Waltham, MA, USA) and resuspended to a concentration of 1.0 × 10^6^ cells per mL for the following experiment. To distinguish the HUVEC’s migration from proliferation, 5 μg/mL of Mitomycin-C was added into the cell medium and incubated for 2 h before CFZ’s creation [44]. The cisplatin (Wuhan Fortuna Chemical Co., Ltd., Wuhan, China) and doxorubicin (Shanghai Macklin Biochemical Co., Ltd., Shanghai, China) used in the cell migration assay were diluted with a solvent according to their product instructions.

Dead staining of cells was performed by using the live/dead double-staining kit (Abbkine, Wuhan, China) for 30 min according to the product instructions. An inverted fluorescence microscope (Nikon Eclipse TI, Japan) equipped with a DS-U3 digital camera was used for observing experimentation and taking images. Post-processing and analysis of images were assisted with Image-Pro plus software (Media Cybernetics, Rockville, MD, USA).

### 4.3. Experimental Setup of Cell Exclusion and Cell Depletion

**Figure 6:**
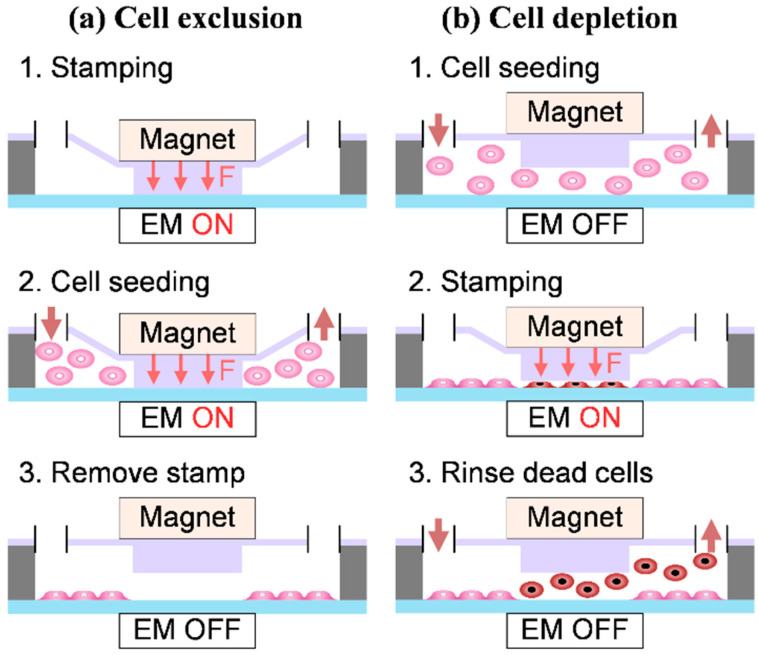
Schematic illustrations of the principles of (**a**) cell exclusion and (**b**) cell depletion in our system. The magnet represents the magnet and the stamp underneath it for the sake of understanding. The switch of the electromagnet module (EM) is controlled by Arduino.

Prior to the experiment, the micro-device was sterilized by 75% ethanol, UV-light exposure overnight, and washed several times with a 5 mL phosphate buffer solution (PBS). By squeezing and washing with liquid, the bubbles generated in the previous steps can be completely discharged. As shown in Figure 6, both methods of cell exclusion (a) and cell depletion (b), leading to the formation of cell patterns, can be realized with the same device. Due to the electromagnetic-controlled stamping process, both methods have the same robustness and reliability and can be used in subsequent applications. Pillars, used as micro-stencils in the cell exclusion method, were forced down with the magnet, which was attracted when the electromagnet module (EM) was turned on. The attraction of the electromagnet was controlled by Arduino until the pillar array completely contacted the bottom of the culture dish.

Next, the prepared 1 mL cell suspension in DMEM was gently loaded into the inlet by the plastic syringe. The chip was placed in the incubator to provide a stable and appropriate condition for cells. Following a 6-hour period of cell adhesion without fluid flow, the magnet moved back to the origin state of EM off and left the cell pattern on the substrate. Then the culture medium was loaded into the chip to wash redundant cell suspension. Subsequently, the DMEM with or without the drug was induced by the perfusion system. After that, images were taken every 6 h to record the cell migration area. The cell depletion method began with cell seeding. After 6 h of incubation for cell attachment, a circular CFZ was mechanically created by the pillars stamping down as EM on for 5 min. Pillars were reset since EM turned off and 3 mL PBS was injected into the chip to wash DMEM. To remove the cells in the circular CFZ, trypsin was gently loaded into the chip and let stand still at room temperature. After 3 min, DMEM was reloaded into the micro-chip to neutralize trypsin and rinse the dead cell. Then the CFZ was obtained, and the cells were ready for cell migration. By these two methods, researchers can adjust the operation sequence on the same chip according to different experimental environments to complete the same experimental purpose, which demonstrates the flexibility of the system in the experiment and application.

### 4.4. Measurement of Stamping Pillars

A series of loading experiments were performed for describing the effect of electromagnetic force generated by the EM system on the deformation of the micro-pillar and the performance of the stamping process. In order to distinguish the contacting surface of stencils or stamps in the cell depletion method from the culture dish substrate under the microscope, the red edible pigment was injected into the micro-device. With the irregular AOI feature in Image-Pro plus software, deformation of the contact surface could be quantified and compared. Meanwhile, the height of the chamber was estimated by the optical intensity of the pigment between the micro-pillars.

### 4.5. Simulation of the Flow Conditions in the Culture Chamber

In order to investigate the shear condition on the bottom of the culture chamber, a set of fluid simulations was performed by the multi-physics simulation software COMSOL Multiphysics 5.3a (COMSOL, Stockholm, Sweden). This software was used to build model geometries, create a mesh, add the physics interfaces and material properties, solve the model, and visualize the results. At first, according to the design drawings of the cavity, the 3D model of the chamber with micro-pillar arrays was built (length = 26 mm, width = 5 mm, and height = 800 μm). For the accuracy and efficiency of operation, the model was divided into two parts which were separately meshed with tetrahedral and hexahedral (block) elements corresponding to the region of micro-pillars and the underside. The laminar flow model was applied to calculate the distribution of shear stress on the bottom, under the influence of micro-pillars. The model used in simulation neglected the lateral movement and deformation of the chamber wall.

### 4.6. Quantification of Cell Migration

Using Adobe Photoshop CC 2018, the conventional or fluorescence pictures were transformed to grayscale. The leading edge of the CFZ was picked by using the magic wand function and the selected area was filled with colors. These processed images were then imported into Image-Pro plus, and the area of the CFZ was determined by AOI tools and measured through the count/size feature after calibration.

## 5. Conclusions

The cell migration assay (device) developed in our work has the following merits: (1) being able to provide different fluidic environments suitable to different types of cells; (2) being able to monitor the movement of cells in many identical cell-free zones in real-time [33]; (3) being able to produce many samples in the monolayer cells under the same condition. The device should, thus, improve the reliability of experiments in two areas: (i) Accuracy of the result due to the fluidic environment where cells are migrating while under stress, and (ii) reproducibility due to the simultaneous production of nearly 400 samples on the same condition. The effectiveness of our device has been demonstrated by two studies related to cell migration, namely combination chemotherapy on HeLa cells and anti-angiogenesis of endothelia cells. Specifically, the study of HeLa cells has found that the response of the cells to drug action in a fluid environment is significantly different from those in a non-fluid condition.

## Data Availability

Data is contained within article.

## References

[1] Kratz S.R.A., Eilenberger C., Schuller P., Bachmann B., Spitz S., Ertl P., Rothbauer M. (2019). Characterization of four functional biocompatible pressure-sensitive adhesives for rapid prototyping of cell-based lab-on-a-chip and organ-on-a-chip systems. Sci. Rep..

[2] Ko Y.G., Co C.C., Ho C.C. (2013). Directing cell migration in continuous microchannels by topographical amplification of natural directional persistence. Biomaterials.

[3] Shirure V.S., Lam S.F., Shergill B., Chu Y.E., Ng N.R., George S.C. (2020). Quantitative design strategies for fine control of oxygen in microfluidic systems. Lab Chip.

[4] Pan Y., Wu Q., Qin L., Cai J., Du B. (2014). Gold nanoparticles inhibit VEGF165-induced migration and tube formation of endothelial cells via the Akt pathway. Biomed. Res. Int..

[5] Ray A., Slama Z.M., Morford R.K., Madden S.A., Provenzano P.P. (2017). Enhanced Directional Migration of Cancer Stem Cells in 3D Aligned Collagen Matrices. Biophys. J..

[6] Ridley A.J., Schwartz M.A., Burridge K., Firtel R.A., Ginsberg M.H., Borisy G., Parsons J.T., Horwitz A.R. (2003). Cell migration: Integrating signals from front to back. Science.

[7] Kim J.H., Serra-Picamal X., Tambe D.T., Zhou E.H., Park C.Y., Sadati M., Park J.A., Krishnan R., Gweon B., Millet E. (2013). Propulsion and navigation within the advancing monolayer sheet. Nat. Mater..

[8] Fu X., Liu G., Halim A., Ju Y., Luo Q., Song A.G. (2019). Mesenchymal Stem Cell Migration and Tissue Repair. Cells.

[9] Si H., Xing T., Ding Y., Zhang H., Yin R., Zhang W. (2019). 3D Bioprinting of the Sustained Drug Release Wound Dressing with Double-Crosslinked Hyaluronic-Acid-Based Hydrogels. Polymers.

[10] Jia Y., Zhang H., Yang S., Xi Z., Tang T., Yin R., Zhang W. (2018). Electrospun PLGA membrane incorporated with andrographolide-loaded mesoporous silica nanoparticles for sustained antibacterial wound dressing. Nanomedicine.

[11] Barbu A., Neamtu B., Zăhan M., Iancu G.M., Bacila C., Mireșan V. (2021). Current Trends in Advanced Alginate-Based Wound Dressings for Chronic Wounds. J. Pers. Med..

[12] Jones E., Marsh S., O’Shaughnessy R., Camera E., Picardo M., Aumailley M., O’Toole E., Caley M. (2019). 274 junctional epidermolysis bullosa: Bottom up control of the skin barrier?. J. Investig. Dermatol..

[13] Buchanan N.D., Grimmer J.A., Tanwar V., Schwieterman N., Mohler P.J., Wold L.E. (2020). Cardiovascular risk of electronic cigarettes: A review of preclinical and clinical studies. Cardiovasc. Res..

[14] Yin L., Du G., Zhang B., Zhang H., Yin R., Zhang W., Yang S.M. (2020). Efficient Drug Screening and Nephrotoxicity Assessment on Co-culture Microfluidic Kidney Chip. Sci. Rep..

[15] Ergir E., Bachmann B., Redl H., Forte G., Ertl P. (2018). Small Force, Big Impact: Next Generation Organ-on-a-Chip Systems Incorporating Biomechanical Cues. Front. Physiol..

[16] Swain S.M., Liddle R.A. (2021). Piezo1 acts upstream of TRPV4 to induce pathological changes in endothelial cells due to shear stress. J. Biol. Chem..

[17] Bai L., Shyy J.Y.P. (2018). Shear Stress Regulation of Endothelium: A Double-edged Sword. J. Transl. Int. Med..

[18] Molladavoodi S., Robichaud M., Wulff D., Gorbet M. (2017). Corneal epithelial cells exposed to shear stress show altered cytoskeleton and migratory behaviour. PLoS ONE.

[19] Yang Y., Zheng H., Zhan Y., Fan S. (2019). An emerging tumor invasion mechanism about the collective cell migration. Am. J. Transl. Res..

[20] Charras G., Sahai E. (2014). Physical influences of the extracellular environment on cell migration. Nat. Rev. Mol. Cell Biol..

[21] Yang S.M., Lin Q., Zhang H., Yin R., Zhang W., Zhang M., Cui Y. (2021). Dielectrophoresis assisted high-throughput detection system for multiplexed immunoassays. Biosens. Bioelectron..

[22] Pijuan J., Barcelo C., Moreno D.F., Maiques O., Siso P., Marti R.M., Macia A., Panosa A. (2019). In vitro Cell Migration, Invasion, and Adhesion Assays: From Cell Imaging to Data Analysis. Front. Cell Dev. Biol..

[23] Liang C.C., Park A.Y., Guan J.L. (2007). In vitro scratch assay: A convenient and inexpensive method for analysis of cell migration in vitro. Nat. Protoc..

[24] Lin J.Y., Lo K.Y., Sun Y.S. (2019). A microfluidics-based wound-healing assay for studying the effects of shear stresses, wound widths, and chemicals on the wound-healing process. Sci. Rep..

[25] Sticker D., Lechner S., Jungreuthmayer C., Zanghellini J., Ertl P. (2017). Microfluidic Migration and Wound Healing Assay Based on Mechanically Induced Injuries of Defined and Highly Reproducible Areas. Anal. Chem..

[26] Shabestani Monfared G., Ertl P., Rothbauer M. (2020). An on-chip wound healing assay fabricated by xurography for evaluation of dermal fibroblast cell migration and wound closure. Sci. Rep..

[27] Vedula S.R., Ravasio A., Lim C.T., Ladoux B. (2013). Collective cell migration: A mechanistic perspective. Physiology.

[28] Riahi R., Yang Y., Zhang D.D., Wong P.K. (2012). Advances in wound-healing assays for probing collective cell migration. J. Lab. Autom..

[29] Gojova A., Barakat A.I. (2005). Vascular endothelial wound closure under shear stress: Role of membrane fluidity and flow-sensitive ion channels. J. Appl. Physiol. (1985).

[30] van der Meer A.D., Vermeul K., Poot A.A., Feijen J., Vermes I. (2010). A microfluidic wound-healing assay for quantifying endothelial cell migration. Am. J. Physiol. Heart Circ. Physiol..

[31] Shih H.C., Lee T.A., Wu H.M., Ko P.L., Liao W.H., Tung Y.C. (2019). Microfluidic Collective Cell Migration Assay for Study of Endothelial Cell Proliferation and Migration under Combinations of Oxygen Gradients, Tensions, and Drug Treatments. Sci. Rep..

[32] Chou S.E., Lee K.L., Wei P.K., Cheng J.Y. (2021). Screening anti-metastasis drugs by cell adhesion-induced color change in a biochip. Lab Chip.

[33] Poujade M., Grasland-Mongrain E., Hertzog A., Jouanneau J., Chavrier P., Ladoux B., Buguin A., Silberzan P. (2007). Collective migration of an epithelial monolayer in response to a model wound. Proc. Natl. Acad. Sci. USA.

[34] Montiel M., Urso L., de la Blanca E.P., Marsigliante S., Jimenez E. (2009). Cisplatin reduces endothelial cell migration via regulation of type 2-matrix metalloproteinase activity. Cell. Physiol. Biochem..

[35] Jones E.M., Cochrane C.A., Percival S.L. (2015). The effect of pH on the extracellular matrix and biofilms. Adv. Wound Care.

[36] Lee Y.J., Chang W.W., Chang C.P., Liu T.Y., Chuang C.Y., Qian K., Zheng Y.G., Li C. (2019). Downregulation of PRMT1 promotes the senescence and migration of a non-MYCN amplified neuroblastoma SK-N-SH cells. Sci. Rep..

[37] White F.M. (2006). Viscous Fluid Flow.

[38] Bagri A., Kouros-Mehr H., Leong K.G., Plowman G.D. (2010). Use of anti-VEGF adjuvant therapy in cancer: Challenges and rationale. Trends Mol. Med..

[39] Shirmanova M.V., Druzhkova I.N., Lukina M.M., Dudenkova V.V., Ignatova N.I., Snopova L.B., Shcheslavskiy V.I., Belousov V.V., Zagaynova E.V. (2017). Chemotherapy with cisplatin: Insights into intracellular pH and metabolic landscape of cancer cells in vitro and in vivo. Sci. Rep..

[40] Laurencot C.M., Kennedy K.A. (1995). Influence of pH on the cytotoxicity of cisplatin in EMT6 mouse mammary tumor cells. Oncol. Res..

[41] Yuan C., Tony A., Yin R., Wang K., Zhang W. (2021). Tactile and thermal sensors built from carbon–polymer nanocomposites—A critical review. Sensors.

[42] Barbu A., Neamțu M.B., Zăhan M., Mireșan V. (2020). Trends in alginate-based films and membranes for wound healing. Rom. Biotechnol. Lett..

[43] Papaioannou T.G., Stefanadis C. (2005). Vascular wall shear stress: Basic principles and methods. Hell. J. Cardiol..

[44] Shikatani E.A., Trifonova A., Mandel E.R., Liu S.T., Roudier E., Krylova A., Szigiato A., Beaudry J., Riddell M.C., Haas T.L. (2012). Inhibition of proliferation, migration and proteolysis contribute to corticosterone-mediated inhibition of angiogenesis. PLoS ONE.

